# Comparison of Volatile Oil between the *Ligusticum sinese* Oliv. and *Ligusticum jeholense* Nakai et Kitag. Based on GC-MS and Chemical Pattern Recognition Analysis

**DOI:** 10.3390/molecules27165325

**Published:** 2022-08-21

**Authors:** Shengmao Li, Yu Huang, Fan Zhang, Hui Ao, Lu Chen

**Affiliations:** 1 School of Pharmacy, North Sichuan Medical College, Nanchong 637100, China; 2 College of Pharmacy, Chengdu University of Traditional Chinese Medicine, Chengdu 611137, China

**Keywords:** *Ligusticum sinese* Oliv., *Ligusticum jeholense* Nakai et Kitag., volatile oils, gas chromatography-mass spectrometry (GC-MS), chemical pattern recognition, species identification

## Abstract

*Ligustici Rhizoma* et Radix (LReR) is the dried rhizomes and roots of *Ligusticum sinese* Oliv. (LS) or *Ligusticum jeholense* Nakai et Kitag. (LJ). However, in the market, LS and LJ are frequently confused with each other. Since the volatile oils are both the main active components and quality control indicators of LReR, a strategy combining gas chromatography-mass spectrometry (GC-MS) and chemical pattern recognition (CPR) was used to compare the volatile components of LJ and LS. Total ion chromatography (TIC) revealed that phthalides (i.e., neocnidilide) and phenylpropanoids (i.e., myristicin) could be thought of as the most critical components in the volatile oils of LJ and LS, respectively. In addition, the chemical components of the volatile oils in LJ and LS were successfully distinguished by hierarchical cluster analysis (HCA) and principal component analysis (PCA). Moreover, two quality markers, including myristicin and neocnidilide, with a very high discriminative value for the classification of LJ and LS, were found by orthogonal partial least squares discriminant analysis (OPLS-DA). The relative contents of myristicin and neocnidilide were 10.86 ± 6.18% and 26.43 ± 19.63% for LJ, and 47.43 ± 12.66% and 2.87 ± 2.31% for LS. In conclusion, this research has developed an effective approach to discriminating LJ and LS based on volatile oils by combining GC-MS with chemical pattern recognition analysis.

## 1. Introduction

**Figure 1:**
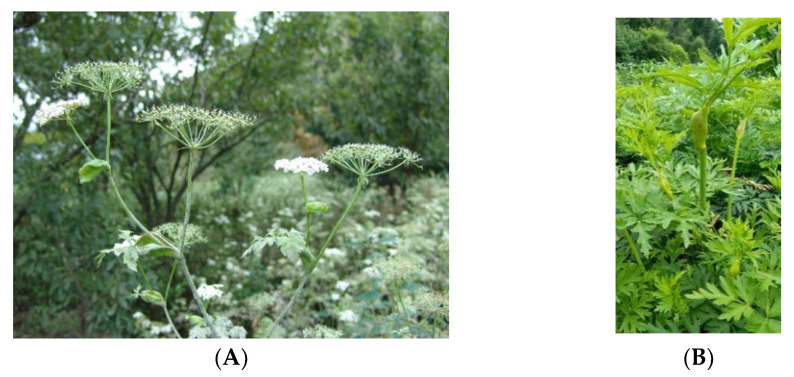
The photos of (**A**) *Ligusticum sinese* Oliv. or (**B**) *Ligusticum jeholense* Nakai et Kitag.

*Ligustici Rhizoma* et Radix (LReR), also known as Gaoben, was first recorded in Shen Nong’s Classic of Materia Medica, which has been used in treating wind chill headaches and rheumatic arthralgia in China for more than one thousand years [1]. Currently, in clinical TCM, more than one hundred Chinese patent medicines use LReR as a primary raw material [2]. Two species are used as LReR, including the dried rhizomes and roots of *Ligusticum sinese* Oliv. (LS) or *Ligusticum jeholense* Nakai et Kitag. (LJ) (Figure 1) [3]. Notably, compared to LJ, LS is generally considered to have a better quality [4], and both are frequently confused with each other in the herbal market. Moreover, modern pharmacological studies showed that LS’s anti-inflammatory [2] and vasodilator effects [5] were more potent than LJ’s. Hence, the differences in biological activities between the two during clinical applications should be noted. In fact, as both are derived from the roots and rhizomes of the *Ligusticum Umbelliferae* family, their outer morphological and internal microstructure features are incredibly similar and difficult to distinguish. Therefore, it is essential to identify the differences between those two species and to discover valuable markers for species identification.

To date, the studies that differentiate LJ from LS are still scant. Several chromatography methods, such as Ultraviolet-visible (UV-Vis) spectroscopy [6,7], Fourier transform infrared spectroscopy (FTIR) [6], thin layer chromatography (TLC) [6,7], gas chromatography-mass spectrometry (GC-MS) [8], and ultra-high performance liquid chromatography quadruple time-of-flight mass spectrometry (UHPLC-QTOF/MS) [9], were performed to identify LReR and its adulterants, respectively, but were unable to distinguish between LJ and LS. Ultra-high performance liquid chromatography (UHPLC) coupled with chemometrics [10] and accD-ycf4 fragments [11] was proven to efficiently discriminate between LJ and LS. However, the former cannot provide enough discriminative information, such as chemical markers. The latter has a higher demand on DNA. It is challenging to extract high-quality DNA because LReR is the dried rhizomes and roots, and a large amount of DNA will be degraded and destroyed during the lengthy drying process. Therefore, the DNA barcode is poorly suited for identifying dried LReR.

LReR is abundant with volatile oils and contains small amounts of non-volatile substances such as organic acids and alkaloids. The volatile oil from LReR mainly contained myristicin, and phthalides represented by ligustilide were one of the main active components in LReR that possessed not only anti-inflammatory and vasodilatory activities, as mentioned above, but also had antithrombotic, analgesic, sedative, antioxidant and anti-tumor effects [11,12,13]. Consequently, the LReR volatile oils and some major components were considered as potential therapeutics for cardiovascular diseases, Alzheimer’s disease, etc. [14,15]. As a result, the volatile components are the crucial indexes of LReR quality. However, there is very little information about the activity of non-volatile ingredients in LReR. Distinguishing LJ and LS will be of great use for developing a practical approach to discriminate between them based on the volatile components. However, no such studies relating to the differences in volatile substances between LJ and LS have been reported.

Due to the high complexity and variability of the chemical components of TCM, it was difficult to differentiate among cultivars via a conventional intuitive comparison of the chemical composition. Chemical pattern recognition (CPR) is a multivariate analysis technique that can reveal the law behind the measurement data and that shows significant advantages in differentiating varieties via the analysis and visualization of high-dimensional data [16]. A strategy combining chemical composition analysis with pattern recognition, particularly hierarchical cluster analysis (HCA), principal component analysis (PCA), and orthogonal partial least squares discriminant analysis (OPLS-DA), has been extensively used in the species identification and quality evaluation of TCM in recent years [17,18], and it may be a powerful tool to resolve these problems.

Therefore, in the present study, a strategy that combined GC-MS analysis and pattern recognition was first developed to distinguish LJ and LS. Specifically, GC-MS analysis combined with pattern recognition techniques including HCA and PCA were used to identify these two confounders’ volatile oils. OPLS-DA was employed to search for chemical markers that could discriminate between these two species of LReR. This study aimed to provide more comprehensive and detailed information on differences in the chemical composition of the volatile oils obtained from the two confused species of LReR by GC-MS.

## 2. Results and Discussion

### 2.1. Fingerprints of LJ and LS

According to total ion chromatography (TIC), the chemical composition between LJ and LS showed some similarities and still some differences. Both the peak number and peak area of LJ from 21 min to 27 min were higher than those of LS. Additionally, the chromatogram of LJ and LS had 7 (peaks 2, 4, 5, 7, 8, 10, and 12) and 11 (peaks 1, 2, 3, 4, 6, 7, 8, 9, 10, 11, and 12) common peaks, respectively. Among those peaks, six were present in both varieties, and the sum of their relative contents ranged from 34.74 to 89.23% (70.16 ± 18.73%) in LJ and 72.04 to 84.52% (79.48 ± 3.13%) in LS.

**Figure 2:**
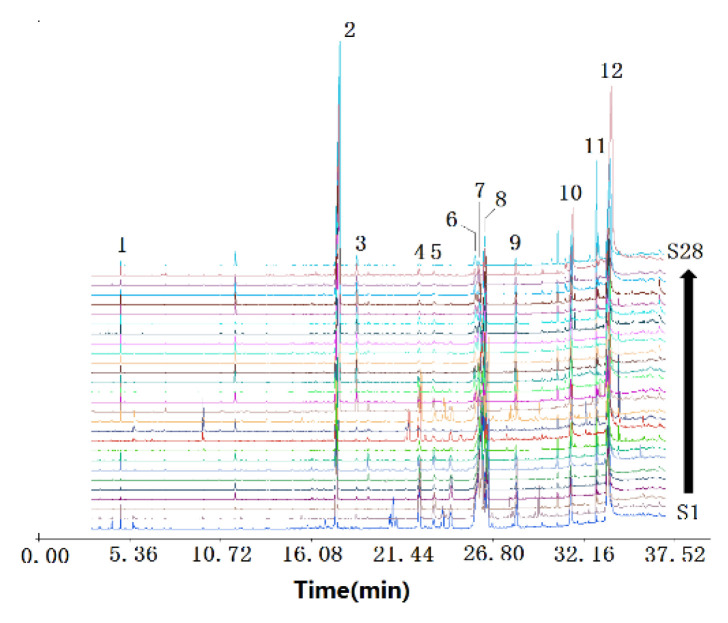
GC-MS chromatogram of 28 batches of *Ligustici Rhizoma et* Radix (LReR) from the two species: (1) β-Phellandrene; (2) Myristicin; (3) Elemicin; (4) 3-Butylisobenzofuran-1(3H)-one; (5) Z-Butylidenephthalide; (6) Senkyunolide A; (7) Neocnidilide; (8) Z-Ligustilide; (9) E-Ligustilide; (10) Palmitic acid; (11) Methyl linoleate; (12) Linoleic acid.

**Figure 3:**
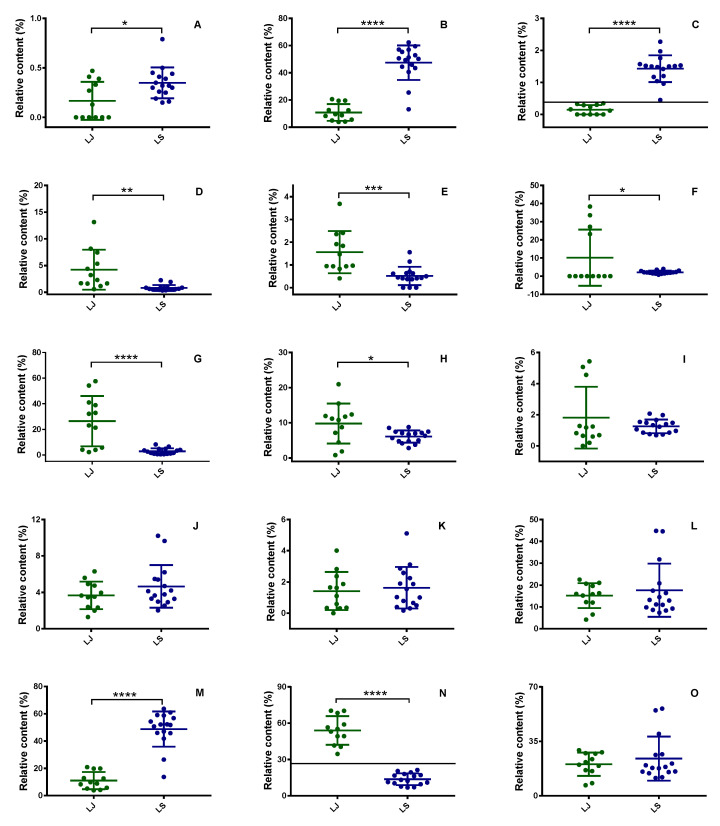
Relative contents of the characteristic composition in total ion chromatography (TIC) of LReR from the two species: (**A**) β-Phellandrene, (**B**) Myristicin, (**C**) Elemicin, (**D**) 3-Butylisobenzofuran-1(3H)-one, (**E**) Z-Butylidenephthalide, (**F**) Senkyunolide A, (**G**) Neocnidilide, (**H**) Z-Ligustilide, (**I**) E-Ligustilide, (**J**) Palmitic acid, (**K**) Methyl linoleate, (**L**) Linoleic acid, (**M**) Total Phenylpropanoids, (**N**) Total phthalides, and (**O**) Total fatty acids; Data were shown as mean ± SEM. **** *p* < 0.0001, *** *p* < 0.001, ** *p* < 0.01, * *p* < 0.05 compared with LReR.

As is known, the species is one of the main factors influencing the chemical composition of herbs, including LReR. Hence, choosing the common peaks that appear in the fingerprints of both LS and LJ for discriminant analysis may lead to missing key information on the chemical composition of one or both species. In order to maintain the relatively complete chemical information on LReR, common peaks of either LJ or LS were selected as the characteristic peaks of LReR for further analysis. As shown in Supplementary Table S1 and Figure 2 and Figure 3, there were 12 characteristic peaks of both species, mainly belonging to terpenoids (peak 1), phenylpropanoids (peaks 2 and 3), phthalides (peaks 4, 5, 6, 7, 8, and 9), and fatty acids (peaks 10, 11, and 12). The sum of their relative peak areas in almost all the samples was more than 80% of the total areas, except for samples S9 (77.29%), S11 (77.16%), and S21 (77.57%). This means that those 12 characteristic components could represent the chemical features of LReR relatively well. The relative contents of the 12 characteristic components were different between the two species of LReR. The highest average relative contents of neocnidilide were detected in LJ, whereas the highest average relative contents of myristicin were found in LS. At the same time, LJ had a significantly higher relative content of neocnidilide compared to LS, and the relative content of myristicin in the LS was significantly higher compared with LJ. In the remaining ten components, the relative contents of β-phellandrene and elemicin in LJ were significantly lower than those in LS. In comparison, the relative contents of 3-butylisobenzofuran-1(3H)-one, Z-butylidenephthalide, senkyunolide A, and Z-ligustilide in LJ were significantly higher than those in LS.

Moreover, phenylpropanoids, phthalides, and fatty acids were the major types of volatile components in both LJ and LS. LJ displayed the highest phthalides (54.01 ± 11.79%) relative contents, which was then followed, in succession, by fatty acids (20.29 ± 7.48%) and phenylpropanoids (11.01 ± 6.28%). In contrast, the relative contents of these components in LS had the following order: phenylpropanoids (48.86 ± 12.93%), fatty acids (23.92 ± 14.12%), and phthalides (13.67 ± 4.86%). Similar to our findings, Leng et al. reported that the volatile oils of both LJ and LS contained a high relative content of phthalides and phenylpropanoids [8].

In this paper, LJ and LS, mostly deriving from the major LReR-producing areas of China, such as Liaoning, Jilin, Sichuan, Chongqing, Hubei, and Shanxi, had been collected, which could reflect the quality characteristics of the mainstream species of LReR in the market. The result shows that neocnidilide and myristicin are the most dominant volatile oil compounds in the LJ and LS, respectively. Neocnidilide possesses antimelanogenesis [19], anti-inflammatory [20], sedative [21], and xanthine oxidase inhibitory [22] activities. Myristicin has anti-inflammatory, analgesic, antiproliferative, antimicrobial, antioxidant, insecticide, and larvicide effects [23]. It is generally believed that LReR is mainly used for its anti-inflammatory and analgesic activities [11,24]. Hence, the better quality of LS over LJ may be related to the high amounts of myristicin. Moreover, other phthalides, such as senkyunolide A, Z-butylidenephthalide, Z-ligustilide, and E-ligustilide, were also present in relatively high amounts, although their amounts differed between LJ and LS. Several studies show that phthalides exhibit diverse biological activities, such as cardiovascular protection [15,25], anti-inflammatory [15,26], analgesic [15], antioxidant, antitumor [26], neuroprotective [26,27], nephroprotective [28], and insecticidal [29] activities. Nevertheless, it is unclear whether the difference in pharmacological activities [2,5] and quality [4] between LJ and LS has also been linked to those ingredients, and further research is required.

### 2.2. HCA

**Figure 4:**
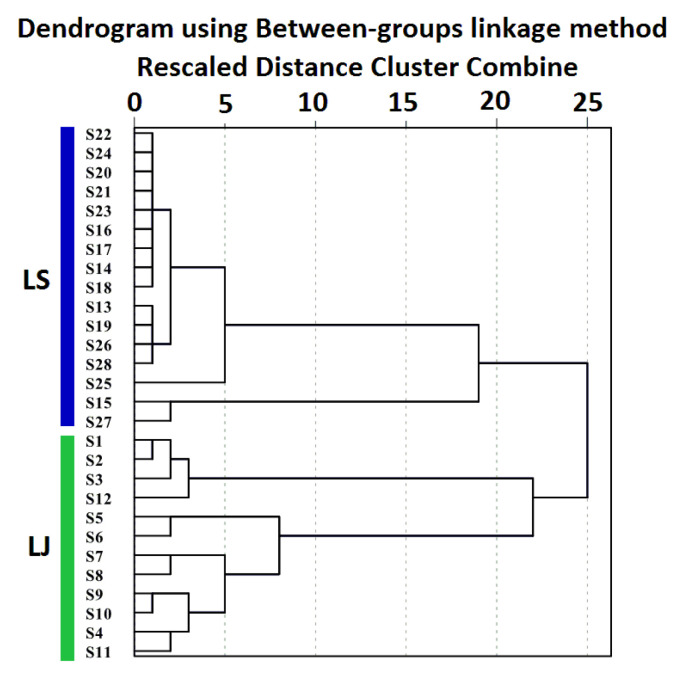
HCA dendrogram of 28 LReR samples from the two species using the Between-groups linkage method based on Squared Euclidean distance.

As an unsupervised pattern recognition method, HCA classifies samples according to their degree of similarity based on the features of the variable and is mostly used for sample groups with no clear classification [30]. In order to find out the objective categories in the patterns of LReR, the relative contents of 12 characteristic components in the essential oil were analyzed using HCA, with the parameter setting for “Between-groups linkage” and “Squared Euclidean distance.” The 28 samples of LReR were segregated into two classes: class I contained S1~S12 (LJ) and class II included S13~S28 (LJ) when the distance scale was about 22. The result is shown in Figure 4. This demonstrated that LJ and LS could be effectively distinguished by the HCA model based on those 12 characteristic components of the oil.

### 2.3. PCA

**Figure 5:**
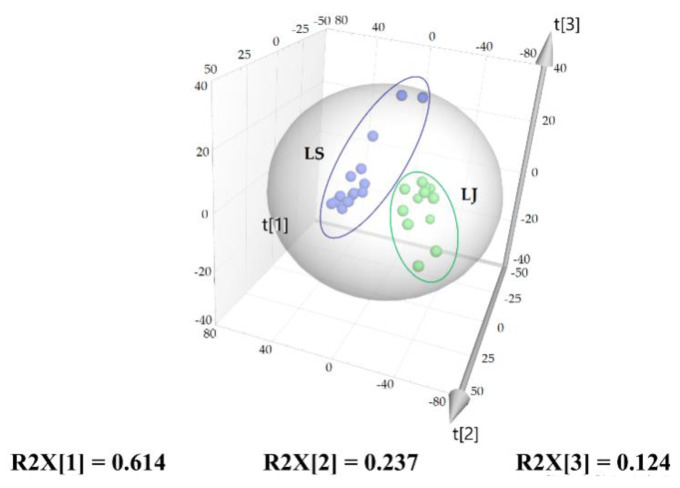
Score plot of PCA of 28 samples of LReR from the two species.

PCA is an unsupervised pattern recognition technique that can cluster and visualize high-dimensional data through linear dimensionality reduction [31,32,33]. After the initial data centering, the relative contents of 12 characteristic components were used as the variables in the PCA model. Three principal components (PCs) were extracted with eigenvalues greater than 1.0, and they explained 97.6% (PC1, 61.4%; PC2, 23.7%; PC3, 12.4%) of the original 12 variables. The Q2 value was 0.778, representing a good predictive power of this model. A three-dimensional (3D) scatterplot was constructed based on the first three PCs. As shown in Figure 5, LJ (S1–S12) and LS (S13–S28) were observed to be distributed in two different regions, which was identical to the HCA results.

### 2.4. OPLS-DA

**Figure 6:**
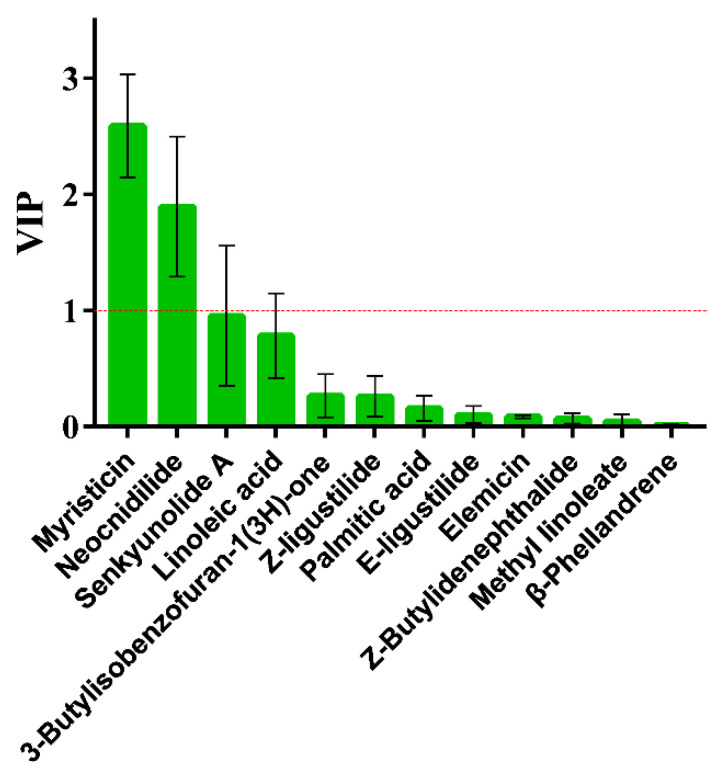
VIP values of the 12 characteristic components of 28 samples of LReR from the two species, based on OPLS-DA.

Although HCA and PCA were able to distinguish well between LJ and LS, both could not clearly identify the influence of a variable on sample classification. To further clarify the differences between LJ and LS and find out the important variables (key markers) for species classification, OPLS-DA, a supervised multivariate analysis technique, was used to analyze the relative contents of 12 characteristic components. Variable importance for the projection (VIP) values were used to evaluate how much each component contributed to the separation between groups. The larger the VIP value is, the greater the contribution to the sample classifications is. In this paper, the component with the VIP value > 1 was selected as the key marker for the sample classification [34]. The R2X, R2Y, and Q2 (cum) values of the OPLS-DA model were 0.971, 0.906, and 0.868, which suggested the good fitness and predictive ability of this model. The 12 characteristic components were sorted in descending order according to their VIP values, as illustrated in Figure 6. Among them, two components with VIP values > 1, including myristicin and neocnidilide, were regarded as the chemical markers for distinguishing the two species of LReR. It is worth noting that myristicin and neocnidilide had the highest average relative content in LS and LJ, respectively, and also showed an excellent value for discriminating LJ from LS by an unpaired *t*-test. Considering how the activities of the volatile oils of LReR vary with different species, it is necessary to further investigate whether these differences in activities are caused by the above components.

## 3. Methods

### 3.1. Plants Materials

A total of 28 batches of LReR, including 12 batches of LJ (S1–S12) and 16 batches of LS (S13–S28), were bought from Hehuachi Professional Market for Chinese Herbal Medicine (Chengdu, China) and Anguo Chinese herbal medicine market (Anguo, China). All samples were authenticated by Associate Professor Lu Chen (Chengdu University of TCM) and were deposited at the Laboratory Center of Pharmacy (North Sichuan Medical College). Sample information is shown in Table 1.

### 3.2. Solvents and Chemicals

Both n-hexane and anhydrous sodium sulfate were analytical grade and provided by Sinopharm Chemical Reagent Co., Ltd.(Shanghai, China). Analytical grade petroleum ether (60–90 °C boiling range) was provided by Chengdu Kelong Chemical Co., Ltd. (Chengdu, China).

### 3.3. Extraction of Volatile Oils

Ten grams of the LJ or LS powder were put into a Soxhlet extractor and extracted with 120 mL petroleum ether for 6 h. Then, the volatile oil of LReR (LVO) was generated by removing the petroleum ether via reduced pressure distillation. Twenty milligrams of the oil were weighed and dissolved in 1 mL n-hexane. After that, the solution was filtered through a 0.22 μm filter before GC-MS analysis.

### 3.4. GC-MS Analysis

GC-MS analysis of LVO was performed using Agilent 7890A-5975C GC–MS with an HP-5 MS capillary column (30 m × 0.25 mm × 0.25 µm). The injection volume was 1 μL with a 100:1 (*v*/*v*) ratio split mode. The carrier gas was Helium (99.999% purity, 1 mL/min). The temperature of the injector and interface was set to 250 ℃ and 280 ℃, respectively. The initial oven temperature was kept at 60 ℃ for 2 min, and then it was gradually raised to 110 ℃ at 30 ℃/min, held for 3 min, raised to 150 ℃ at 3 ℃/min, and kept for 5 min. Finally, it was raised to 260 ℃ at 10 ℃/min and kept for 1 min. The mass spectrometer was operated at 70 ev in full scan mode. The compounds in LVO were identified through the NIST14 database (National Institute of Standards and Technology). The area normalization method calculated the relative content of each compound in the chromatogram.

### 3.5. Data Analysis

Statistical analysis was carried out using an unpaired *t*-test by GraphPad Prism 7 (GraphPad Software Inc., La Jolla, CA, USA). Moreover, these data were also analyzed and processed by HCA, PCA, and OPLS-DA using SPSS13.0 (SPSS Inc., Chicago, IL, USA) or SIMCA P14.0 (Umetrics, Umea, Sweden). The results were expressed in means ± SEM, and the level of *p* < 0.05 was considered statistically significant.

## 4. Conclusions

This study established an efficient method for discriminating two species of LReR based on GC-MS fingerprints and chemical pattern recognition analysis.

The GC-MS fingerprint for the two species of LReR shows some similarities and still some differences. Specifically, the main components and the major types of components in the volatile oils were different. LJ presented the highest average relative contents of neocnidilide (2.27–57.60%) and phthalides (34.62–70.28%), while LS had the highest average relative contents of myristicin (13.25–62.28%) and phenylpropanoids (13.70–63.70%). The other six components, including β-phellandrene, elemicin, 3-butylisobenzofuran-1(3H)-one, Z-butylidenephthalide, senkyunolide A, and Z-ligustilide, can also be employed as potential distinguishing components by using the unpaired *t*-test. Moreover, the chemical pattern recognition analysis, such as HCA and PCA based on the GC-MS, revealed significant differences in the volatile composition between LJ and LS. The samples were grouped by species. Additionally, two markers, including myristicin and neocnidilide, with high discriminative values for the classification of LJ and LS were found in OPLS-DA.

In summary, GC-MS fingerprints coupled with chemical pattern recognition analysis could be considered an effective approach for discriminating between LJ and LS.

## Figures and Tables

**Table 1:** Sample information of 28 batches of LReR.

NO.	Origin	Batch Code	NO.	Origin	Batch Code
S1	Benxi, Liaoning	2018001LJ	S15	Kangding, Sichuan	2018003LS
S2	Benxi, Liaoning	2018002LJ	S16	Lixian, Sichuan	2018004LS
S3	Fushun, Liaoning	2018003LJ	S17	Nanchuan, Chongqing	2018005LS
S4	Fushun, Liaoning	2018004LJ	S18	Wushan, Chongqing	2018006LS
S5	Fushun, Liaoning	2018005LJ	S19	Lichuan, Hubei	2018007LS
S6	Yingkou, Liaoning	2018006LJ	S20	Shennongjia, Hubei	2018008LS
S7	Anshan, Liaoning	2018007LJ	S21	Shennongjia, Hubei	2018009LS
S8	Yongji, Jinlin	2018008LJ	S22	Badong, Hubei	2018010LS
S9	Antu, Jilin	2018009LJ	S23	Enshi, Hubei	2018011LS
S10	Antu, Jilin	2018010LJ	S24	Longxian, Shanxi	2018012LS
S11	Changbaishan, Jilin	2018011LJ	S25	Zhenping, Shanxi	2018013LS
S12	Chengde, Hebei	2018012LJ	S26	Longxian, Shanxi	2018014LS
S13	Hanyuan, Sichuan	2018001LS	S27	Lushi, Henan	2018015LS
S14	Guangyuan, Sichuan	2018002LS	S28	Suichuan, Jiangxi	2018016LS

## Data Availability

The data are contained in this article.

## Supplementary Materials

The following supporting information can be downloaded at: https://www.mdpi.com/article/10.3390/molecules27165325/s1, Table S1: Relative contents of characteristic components of *Ligustici Rhizoma* et Radix (LReR) from the two species.

## Sample Availability

Samples of the compounds are not available from the authors.
