# Supplementary material for: Comparison of Volatile Oil between the Ligusticum sinese Oliv. and Ligusticum jeholense Nakai et Kitag. Based on GC-MS and Chemical Pattern Recognition Analysis
Source: Molecules. 2022 Aug 21;27(16):5325. doi: 10.3390/molecules27165325 (PMC9414267; doi:10.3390/molecules27165325)
Supplement: Supplementary file 1 [file molecules-27-05325-s001.zip › molecules-1828610-supplementary.pdf]

Table S1. Relative contents of characteristic components of *Ligustici Rhizoma et* Radix (LReR) from the two species.

No.	Retention Time	Molecular Weight	Component	Molecular Formula	S1	S2	S3	S4	S5	S6	S7	S8	S9	S10	S11	S12	S13	S14	S15	S16	S17	S18	S19	S20	S21	S22	S23	S24	S25	S26	S27	S28	Average relative contents in LJ	Average relative contents in LS
1	4.82	136.2	β-Phellandrene	$C_{10}H_{16}$	0.27	0.39	0.00	0.13	0.00	0.00	0.33	0.41	0.00	0.00	0.00	0.47	0.79	0.26	0.19	0.50	0.30	0.32	0.45	0.41	0.32	0.35	0.44	0.39	0.16	0.25	0.30	0.15	0.17±0.19	0.35±0.16
2	17.77	192.2	Myristicin	$C_{11}H_{12}O_3$	5.63	12.51	8.30	19.60	12.63	4.17	9.65	19.52	8.80	4.95	20.63	3.92	43.53	62.28	13.25	57.10	56.99	59.53	44.50	52.87	50.64	50.23	55.37	49.27	40.62	45.89	25.49	51.28	10.86±6.18	47.43±12.66
3	18.75	208.2	Elemicin	$C_{12}H_{16}O_3$	0.12	0.35	0.00	0.33	0.00	0.00	0.16	0.29	0.00	0.24	0.30	0.00	2.27	1.42	0.45	1.97	1.76	1.53	1.50	1.52	1.50	1.57	1.48	1.51	1.20	1.17	0.96	1.04	0.15±0.15	1.43±0.42
4	22.42	190.2	3-Butylisobenzofuran-1(3H)-one	$C_{12}H_{14}O_2$	4.38	5.34	13.14	1.69	1.61	3.22	1.64	1.15	2.29	7.44	0.57	8.15	2.25	0.54	1.93	0.85	0.45	0.50	0.91	0.65	0.49	0.94	0.59	0.62	0.33	0.55	0.84	0.36	4.22±3.74	0.80±0.54
5	23.30	188.2	Z-Butylidenephthalide	$C_{12}H_{12}O_2$	0.95	1.92	3.69	0.97	0.83	1.47	2.36	2.41	0.94	0.94	0.41	1.84	1.56	0.37	1.15	0.41	0.38	0.43	0.71	0.64	0.45	0.63	0.41	0.50	0.00	0.00	0.62	0.00	1.56±0.93	0.52±0.40
6	25.74	192.2	Senkyunolide A	$C_{12}H_{16}O_2$	33.59	27.20	23.24	0.00	0.00	0.00	0.00	0.00	0.00	0.00	0.00	38.38	4.01	1.85	3.56	1.88	1.30	2.20	2.87	2.05	1.47	3.05	2.11	2.34	1.39	1.94	0.81	1.50	10.20±15.47	2.15±0.86
7	26.02	194.2	Neocnidilide	$C_{12}H_{18}O_2$	2.27	3.95	5.71	41.02	54.20	57.60	23.12	21.28	32.18	38.91	32.85	4.12	3.50	0.99	4.74	0.64	1.72	0.87	8.21	6.48	1.79	3.98	3.41	4.84	0.47	2.62	1.13	0.58	26.43±19.63	2.87±2.31
8	26.38	190.2	Z-Ligustilide	$C_{12}H_{14}O_2$	10.78	11.75	11.94	8.73	11.19	7.16	20.96	15.47	4.40	1.85	0.79	12.36	7.14	5.65	4.15	4.74	6.31	6.97	7.08	6.72	7.48	8.79	8.55	7.47	4.77	5.01	2.80	3.73	9.78±5.69	6.09±1.74
9	28.15	190.2	E-Ligustilide	$C_{12}H_{14}$O2	4.56	5.07	1.28	0.65	0.60	0.82	1.18	1.24	0.69	0.20	0.00	5.43	2.07	0.96	0.75	1.06	1.23	1.33	1.54	1.48	1.48	1.98	1.66	1.40	0.73	0.85	0.85	0.68	1.81±1.98	1.25±0.44
10	31.36	256.4	Palmitic acid	$C_{16}H_{32}$O2	3.65	3.52	5.60	2.33	1.29	2.39	3.96	3.46	4.92	6.29	4.75	1.99	2.02	2.98	10.21	4.70	4.21	3.31	5.41	3.78	3.29	2.92	2.55	4.14	6.19	5.48	9.64	3.66	3.68±1.52	4.66±2.34
11	32.89	294.4	Methyl linoleate	$C_{19}H_{34}O_2$	0.31	0.35	1.63	1.10	0.33	1.87	2.82	4.01	2.37	0.00	1.65	0.59	0.17	2.86	1.01	1.03	2.58	3.11	0.79	0.50	0.31	1.87	1.55	2.25	1.90	0.39	0.66	5.11	1.42±1.22	1.63±1.32
12	33.56	280.4	Linoleic acid	$C_{18}H_{32}O_2$	15.99	12.01	16.32	15.86	6.51	12.24	22.55	19.63	20.70	21.08	15.21	4.20	16.38	8.65	44.83	9.81	11.14	9.25	14.53	10.98	8.35	12.94	7.37	13.23	31.73	20.84	44.62	17.49	15.19±5.69	17.63±12.16
			Total percentages of identified components		88.59	90.26	96.06	96.96	96.53	96.87	93.82	96.78	87.85	88.01	88.88	89.73	95.37	98.32	95.58	96.10	96.92	98.94	98.4	95.9	93.88	95.30	94.40	97.42	98.35	96.90	97.64	96.81		
			Total percentages of characteristic components		82.50	84.36	90.85	92.41	89.19	90.94	88.73	88.87	77.29	81.90	77.16	81.45	85.69	88.81	86.22	84.69	88.37	89.35	88.50	88.08	77.57	89.25	85.49	87.96	89.49	84.99	88.72	85.58		
			Total percentages of phenylpropanoids		5.75	12.86	8.30	19.93	12.63	4.17	9.81	19.81	8.80	5.19	20.93	3.92	45.80	63.70	13.70	59.07	58.75	61.06	46.00	54.39	52.14	51.80	56.85	50.78	41.82	47.06	26.45	52.32		
			Total percentages of phthalides		56.53	55.23	59.00	53.06	68.43	70.27	49.26	41.55	40.50	49.34	34.62	70.28	20.53	10.36	16.28	9.58	11.39	12.30	21.32	18.02	13.16	19.37	16.73	17.17	7.69	10.97	7.05	6.85		
			Total percentages of fatty acids		19.95	15.88	23.55	19.29	8.13	16.50	29.33	27.10	27.99	27.37	21.61	6.78	18.57	14.49	56.05	15.54	17.93	15.67	20.73	15.26	11.95	17.73	11.47	19.62	39.82	26.71	54.92	26.26		
